# Optimizing photo-Fenton like process for the removal of diesel fuel from the aqueous phase

**DOI:** 10.1186/2052-336X-12-87

**Published:** 2014-05-23

**Authors:** Mansooreh Dehghani, Esmaeel Shahsavani, Mahdi Farzadkia, Mohammad Reza Samaei

**Affiliations:** 1 Department of Environmental Health Engineering, School of Health, Shiraz University of Medical Sciences, Shiraz, Iran; 2 Department of Environmental Health Engineering, Iran University of Medical Sciences, Tehran, Iran

**Keywords:** Photo-Fenton like process, nZVI, Taguchi method, TPH, Diesel fuel

## Abstract

**Background:**

In recent years, pollution of soil and groundwater caused by fuel leakage from old underground storage tanks, oil extraction process, refineries, fuel distribution terminals, improper disposal and also spills during transferring has been reported. Diesel fuel has created many problems for water resources. The main objectives of this research were focused on assessing the feasibility of using photo-Fenton like method using nano zero-valent iron (nZVI/UV/H_2_O_2_) in removing total petroleum hydrocarbons (TPH) and determining the optimal conditions using Taguchi method.

**Results:**

The influence of different parameters including the initial concentration of TPH (0.1-1 mg/L), H_2_O_2_ concentration (5-20 mmole/L**)**, nZVI concentration (10-100 mg/L), pH (3-9), and reaction time (15-120 min) on TPH reduction rate in diesel fuel were investigated. The variance analysis suggests that the optimal conditions for TPH reduction rate from diesel fuel in the aqueous phase are as follows: the initial TPH concentration equals to 0.7 mg/L, nZVI concentration 20 mg/L, H_2_O_2_ concentration equals to 5 mmol/L, pH 3, and the reaction time of 60 min and degree of significance for the study parameters are 7.643, 9.33, 13.318, 15.185 and 6.588%, respectively. The predicted removal rate in the optimal conditions was 95.8% and confirmed by data obtained in this study which was between 95-100%.

**Conclusion:**

In conclusion, photo-Fenton like process using nZVI process may enhance the rate of diesel degradation in polluted water and could be used as a pretreatment step for the biological removal of TPH from diesel fuel in the aqueous phase.

## Introduction

In recent years, pollution of soil and groundwater caused by fuel leakage from old underground storage tank, refineries, fuel distribution terminals, improper disposal and also spills during transferring, has been reported [1-3]. The accidental spills of more than 2 million tons of refined oil products per year into the environment are a worldwide problem [4]. Large amounts of benzene, toluene, ethyl benzene, and xylenes (BTEX) have been detected in polluted water resources [5]. The leaching of petroleum hydrocarbons such as diesel fuel into water resources causes many serious environmental problems [6]. Diesel fuel consists of a complex compound including paraffin, olefins, aliphatic hydrocarbons, as well as a lesser amount of aromatic compounds and includes molecules containing sulfur, nitrogen and metal oxides [2]. The toxicity of diesel fuel is mostly due to BTEX aromatic hydrocarbons. The carcinogen property of diesel fuel is due to C_10_ and C_20_ alkenes and alkylated benzene. Therefore, developing an efficient method for the removal of diesel fuel from contaminated water resources is very crucial [4]. Different physical, chemical, and biological techniques have been used to degrade the contaminated soils and water [6-10]. The aromatic hydrocarbons with high toxic nature cannot be degraded simply by conventional treatment methods [11]. Most conventional techniques such as evaporation, oil phase separation, filtration, dissolved air flotation, coagulation, flocculation, absorption, and ultrafiltration only transfer pollutions from one media to another [12,13]. Therefore, another method should be developed to remove hydrocarbon pollutions [14]. In other words, these methods do not remove the pollutants, but generally produce highly concentrated wastes in lower volume. In addition, these methods have lower efficiency in removing smaller oil droplets and emulsions [15]. Nowadays, biological and bioremediation techniques are used to treat polluted soils and sewages, but these methods need improvement for enhancing the enzymatic activity in microbial population [16]. Therefore, it is very important to use advanced methods in order to remove oily compounds from groundwater resources. Advanced oxidation processes (AOPs) is an efficient environment-friendly method in which hydroxyl radicals (OH°) are used to oxidize recalcitrant organic pollutants and convert them to harmless end-products such as H_2_O and CO_2_[17,18].

In this study, photo-Fenton like process which is based on electrochemical system using nano zero-valent iron (nZVI) and peroxide hydrogen as reductive and oxidative reagents, respectively, was used. In photo-Fenton process, the formation of ferrous ion reduces the process effectiveness and eventually it will halt the reaction. In photo-Fenton like process (Fe°/UV/H_2_O_2_), ferrous (Fe^+2^) and ferric (Fe^+3^) are formed, respectively. These ions practically enhance the efficiency of the process [19]:

(1) $$Fe^{\circ }+H_{2}O_{2}\to Fe^{2+}+OH^{\circ }+OH^{-}$$

(2) $$Fe^{2+}+H_{2}O_{2}\to Fe^{3+}+OH^{\circ }+OH^{-}$$

(3) $$\mathrm{Organic}\;\mathrm{material}+OH^{\circ }\to \mathrm{Oxidized}\;\mathrm{compound}+H_{2}O$$

Previous studies in Iran have reported soil and water pollution caused by petroleum compounds near Shiraz, Esfahan and Tehran refineries [6]. Since Fars (in Southern part of Iran) enjoys a top rank in oil refinery, storage, and distribution of oil products in the country in recent years, there is a concern regarding the effect of petroleum hydrocarbons in water resources on people's health and the environment. Therefore, the objectives of the study were to (i) evaluate the feasibility of using photo-Fenton like method (nZVI/UV/H_2_O_2_) in removing total petroleum hydrocarbons (TPH) and (ii) determine the optimal conditions using Taguchi method so that the standard limit can be achieved by further complementary treatment.

## Materials and methods

### Chemicals and analytical method

In this research, regular diesel fuel from a gas station in Shiraz was used as a pollutant model. Water-oil emulsion was prepared using emulsifier SDS. Nano zero-valent iron (nZVI) was supplied by Iran Oil Industry Research Center. The rest of the chemicals were purchased from Merck (Germany). UV lamp, 125 Kw, 247.3 nm wave length, (ARDA, Netherland) was used as the radiation source.

**Figure 1:**
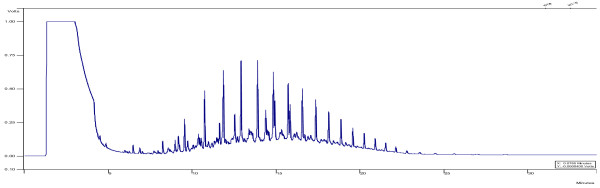
GC chromatograms for the initial sample of diesel fuel.

**Figure 2:**
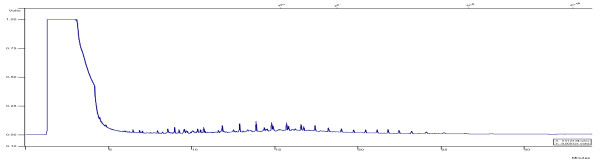
GC chromatograms for the treated sample of diesel fuel.

The standard method No. 8015B of Environment Protection Agency at the United States (EPA) was used to detect petroleum hydrocarbons especially gasoline range organics (GROs, C_6_-C_10_) with the boiling point of 60-170°C, and diesel fuel range organics (DROs, C_10_-C_28_) with the boiling point of 170-430°C [20]. DRO was prepared using liquid-liquid extraction method with n-hexane as solvent [21]. The recovery of the sample from the aqueous phase with this method of extraction was 98%. The method provides the chromatographic conditions for detecting non-halogen volatile organic compounds. Diesel standard was supplied by Merck (Germany, analytical grade GC). For TPH detection in diesel fuel in the aqueous phase a Varian Model gas chromatography (Australia) system with fused silica capillary columns (CP-SIL 5 CB column model, 30 m*0.25 mm, 25 um) was calibrated and tested prior to injection of the samples. A Flame Ionization Detector (FID) was used to detect TPH in the samples. The injector and detector temperatures were set at 200°C and 340°C, respectively. The column temperature was maintained at 45°C for 3 min, and then increased to 275°C (at a rate of 12°C/min), where it was kept for 12 min. The flow rate of helium carrier gas and nitrogen make up gas, were set at 5-7 mL/min and 30 mL/min, respectively. The detection limit for the sample was 0.01 mg/L. GC chromatograms for the initial and treated samples of diesel fuel are presented in Figures 1 and 2.

### Sample preparation

Diesel fuel (commercial grade) at the concentration of 100 mL was added gradually to 1 liter of distilled water using 2.5 mL of 0.1 g/L SDS as emulsifier. The water-diesel emulsion was mixed using Jar test at the speed of 200 rpm for 24 h. In order to separate non-dispersed diesel in oil-water emulsion, the solution was kept at rest for 1 h. Then, the emulsion was transferred to a separator funnel and the supernatant was removed and the rest was collected and passed through Whatman filter paper (20 μm in diameter). The prepared emulsion was used as stock solution and different concentrations 0.1-1 mg/L were prepared by diluting with distilled water.

### Experimental setup

Five parameters including the initial concentration of TPH, H_2_O_2_ concentration, nZVI concentration, pH, and reaction time were selected in 4 levels to analyze the removal efficiency of TPH in diesel fuel in the aqueous phase (Table 1).

**Table 1:** **Parameters and the selected levels of photo-Fenton like treatment process (nZVI/UV/H**_**2**_**O**_**2**_**) for the reduction of TPH from diesel fuel in the aqueous phase**

**Variables level**	**Level 1**	**Level 2**	**Level 3**	**Level 4**
Concentration TPH (mg/L)	0.1	0.4	0.7	1
nZVI Concentration (mg/L)	10	20	40	100
H_2_O_2_ Concentration (mmole/L)	5	10	15	20
pH	3	5	7	9
Reaction time (min)	15	60	90	120

Taguchi’s statistical method and Qualitek-4 (QT4) software were used for the experimental design. Using this software, 16 tests were designed randomly to reduce the errors. Two replications were done for each sample. TPH reduction rate was analyzed using QT4 software. The most effective parameters for removing diesel fuel from the aqueous phase, the rate of efficiency, and the level of precision, and optimal conditions were determined.

### Test conditions and reactor specifications

**Figure 3:**
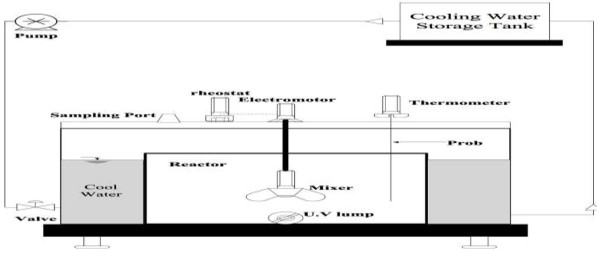
Photochemical reactor.

The specification of photochemical reactor is shown in Figure 3. One liter samples were used in 2 liter volume reactor. Test was performed in a closed reactor with adjustable mixer. The UV radiation source, 1020 μw/Cm^2^, was embedded at the bottom of reactor. The UV lamp was protected by Quartz tube. A thermometer was placed inside the photochemical cell to record the temperature. The temperature inside the reactor was kept at the range of 24-26°C using cooling water recirculation system.

## Results and Discussion

### The effects of nZVI particles concentration

**Figure 4:**
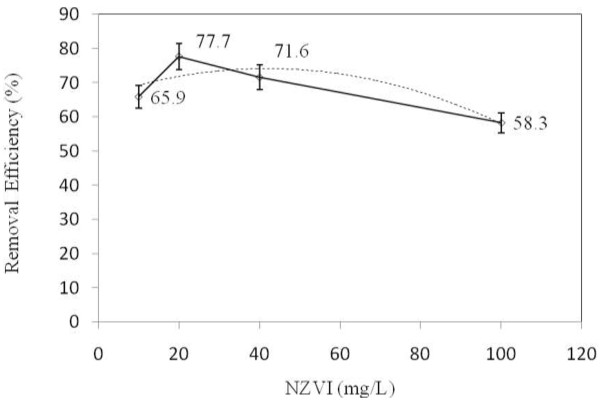
The effect of nZVI on the reduction rate of TPH from diesel fuel in the aqueous phase using photo- Fenton like process.

According to Figure 4, the optimal nZVI concentration and the reduction rate of TPH were 20 mg/L and 77.7%, respectively. The TPH reduction rate in diesel fuel increased from 65.9 to 77.7% as the nZVI concentration increased from 10 to 20 mg/L. However, an increase in the concentration from 20 to 100 mg/L, caused a decrease in the reduction rate of diesel degradation (58.3%). In the current study (nZVI/UV/H_2_O_2_), increasing nZVI increased the production rate of hydroxyl radical. Due to the fact that by increasing nZVI the metal active surface was increased to make better contact with peroxide hydrogen and UV radiation. The reduction rate was decreased by increasing the nZVI concentration above the optimal concentration; in this condition the process is in favor of producing more ferrous ions rather than producing hydroxyl radical [15]. Therefore, TPH reduction rate increased with nZVI concentration up to a specific level (10-20 mg/L) and then began to decrease (20-100 mg/L). The reduction of the pollutant is basically proportional to the formation of hydroxyl radicals on the surface of the catalyst. Due to high turbidity of the solution at higher concentrations of nZVI (20-100 mg/L), UV radiation cannot penetrate into the solution and mostly adsorbed by the particles. In addition, the rate of H_2_O_2_ photolysis depends on the intensity of UV radiation. Therefore, lower UV radiation results in reducing the hydroxyl radical production as well as the degradation rate [20]. The additional Fe ions react with the hydroxyl radical and therefore reduce the efficiency of the process [22].

### pH effect

**Figure 5:**
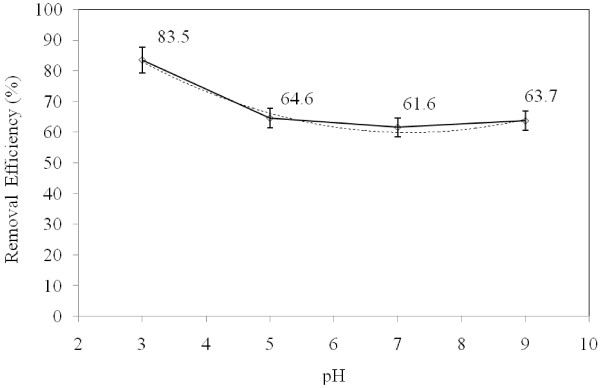
The effect of pH on the reduction rate of TPH from diesel fuel in the aqueous phase using photo- Fenton like process.

Data regarding the effect of pH shows that as pH increased from 3.0 to 9, the rate of TPH reduction decreased. Based on the data obtained in the present study, pH of 3 is optimal for TPH degradation. The reduction rate was more than 83% in this case. Generally, pH is one of the most important factors affecting chemical and biological processes especially advanced oxidation efficiency. In addition, pH has a considerable effect on the solubility of petroleum hydrocarbon, catalyst surface charge, as well as the mechanism of hydroxyl radical production [11]. The Fenton and photo-Fenton reactions depend on the pH. The feasibility of hydroxyl radical production and oxidation efficiency also depend on pH [23]. The reduction rate of TPH reduced in higher pH, because of the formation of ferric hydroxide which in turn reduced the intensity of UV radiation and the potential of hydroxyl radical production as well [24]. Additionally, high pH values intensify the formation of HO_2_^-2^ ions and destruction of hydroxyl radicals by carbonate and bicarbonate ions (Figure 5).

### The effects of reaction time

**Figure 6:**
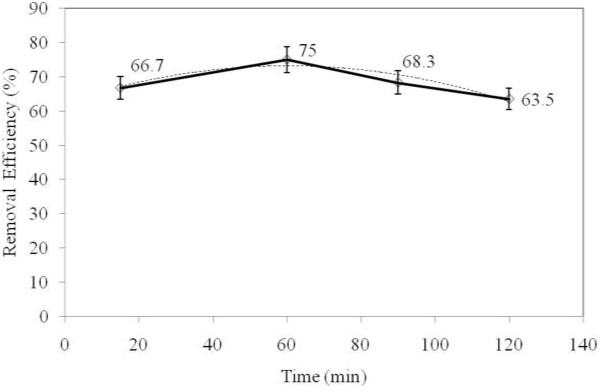
The effect of reaction time on the reduction rate of TPH from diesel fuel in the aqueous phase using photo- Fenton like process.

The effect of reaction time on TPH reduction rate from diesel fuel in the aqueous phase was studied at four levels 15, 60, 90, and 120 min (Figure 6). Data regarding the effect of reaction time shows that as the time increased from 15 to 60 min, the rate of reduction increased by 75%. However, from 60 to 120 min there was a reduction rate in TPH degradation (63.5%). Based on our findings, 60 min reaction time is optimal for TPH degradation (Figure 6). The optimization of reaction time is one of the most important parameters in studying the removal processes. Basically, an optimal contact time is a very important parameter for any chemical reactions. At equilibrium, TPH degradation reached a plateau. If the reaction time exceeds equilibrium, the process will be no longer economical [25]. Coelho et al. showed that the optimal reaction time for the removal of TPH from petroleum wastewater was equal to one hour [26].

### The effect of H_2_O_2_ concentration

**Figure 7:**
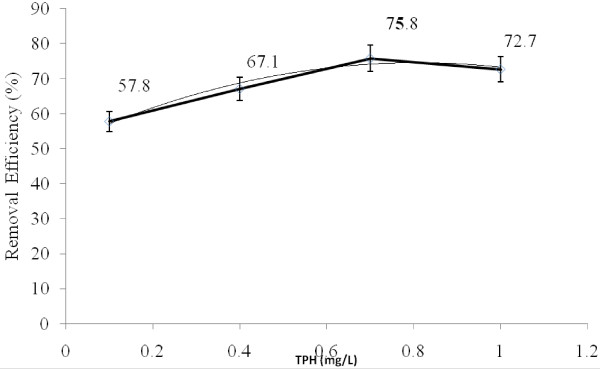
**The effect of H**_**2**_**O**_**2 **_**on the reduction rate of TPH from diesel fuel in the aqueous phase using photo- Fenton like process.**

The effect of H_2_O_2_ concentration on TPH reduction was shown in Figure 7. Based on the data obtained in the present study, H_2_O_2_ concentration of 5 mmol/L is optimal for diesel fuel degradation and the reduction rate was 81.7%. As H_2_O_2_ concentration increased from 5 to 20 mmol/L, TPH reduction rate decreased to 42.5%. Adding extra H_2_O_2_ concentration (more than 5 mmol/L) will act as the scavenger for hydroxyl radical and form HO_2_**°** which has lower oxidative ability and longer lifetime comparing to OH**°**[15]. The decomposition of hydrogen peroxide into oxygen and water occurred at H_2_O_2_ concentration of more than optimal. Therefore, it can be concluded that high concentrations of H_2_O_2_ act as an inhibitor for the formation of hydroxyl radicals' formation and consequently reduced the efficiency of the process [22]. Adding hydrogen peroxide to nZVI considerably reduces production of free electrons and the reaction will mostly tend toward a semi-Fenton process and producing more hydroxyl radicals [26]. Many advanced processes such as ultrasonic and photochemical reaction were used to remove organic pollutants (e.g. diesel) from aqueous solution [27-30].

### The effects of initial TPH concentration

**Figure 8:**
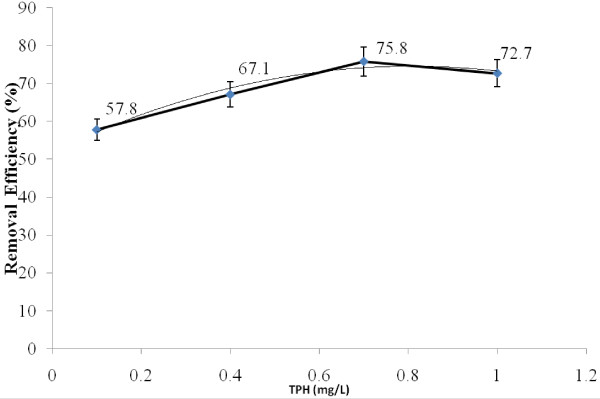
The effect of initial TPH concentration on the reduction rate of TPH from diesel fuel in the aqueous phase using photo- Fenton like process.

The effects of initial TPH concentration on the reduction rate of diesel fuel in the aqueous phase have been shown in Figure 8. As initial TPH concentration of diesel fuel increased from 0.1 to 0.7 mg/L, TPH reduction rate increased from 57.8 to 75.8% (Figure 8). However, as the initial TPH concentration increased from 0.7 to 1.0 mg/L, a decrease in the reduction rate in diesel fuel degradation (72.7%) was seen. The decrease in the TPH reduction rate at more than 0.7 mg/L was possibly the result of increasing the turbidity of the solution and consequently decreasing the UV radiation permeability. Besides, H_2_O_2_ photolysis depends on UV radiation intensity. There is a direct relation between UV radiation, the potential formation of hydroxyl radical and diesel fuel decomposition rate.

## Conclusion

Our results suggest that the photo-Fenton like process (nZVI/UV/H_2_O_2_) can be used as a pre-treatment step for the biological removal of TPH from diesel fuel in the aqueous phase. In the conventional photo-Fenton like methods, high concentrations of ferrous salts were used. Therefore, the large amount of ferrous sludge was formed. The current study revealed that a lower amount of Fe is needed and the nZVI particles can be reused in a magnetic field. The variance analysis suggests that the optimal conditions for TPH reduction rate from diesel fuel in the aqueous phase using photo-Fenton like method (nZVI/UV/H_2_O_2_) are as follows: the initial TPH concentration equals to 0.7 mg/L, nZVI concentration 20 mg/L, H_2_O_2_ concentration equals to 5 mmol/L, pH 3, and the reaction time of 60 min and degree of significance for the study parameters are 7.643, 9.33, 13.318, 15.185 and 6.588 percent, respectively. The predicted removal rate in optimal conditions was 95.8%, confirmed by the results of our study which was between 95-100%.

## Competing interests

The authors declare that they have no competing interests.

## Authors’ contributions

The overall implementation of this study including design, experiments and data analysis, and manuscript preparation were the results of the corresponding author's efforts. All authors have made extensive contribution into the review and finalization of this manuscript. All authors read and approved the final manuscript.
